# Exogenous Application of RNAi-Inducing Double-Stranded RNA Inhibits Aphid-Mediated Transmission of a Plant Virus

**DOI:** 10.3389/fpls.2019.00265

**Published:** 2019-03-15

**Authors:** Elizabeth A. Worrall, Ana Bravo-Cazar, Alexander T. Nilon, Stephen J. Fletcher, Karl E. Robinson, John P. Carr, Neena Mitter

**Affiliations:** ^1^ Centre of Horticultural Science, Queensland Alliance of Agriculture and Food Innovation, The University of Queensland, Brisbane, QLD, Australia; ^2^ Department of Plant Sciences, Cambridge University, Cambridge, United Kingdom

**Keywords:** RNA interference, topical application, double-stranded RNA, potyvirus, nanoparticles, insect vectors

## Abstract

Plant viruses are difficult to control, and they decrease both the quality and yield of crops, thus threatening global food security. A new approach that uses topical application of double-stranded RNA (dsRNA) to induce antiviral RNA-interference has been shown to be effective at preventing virus infection in a range of plants following mechanical inoculation. In this study, topical application of dsRNA was effective against mechanical inoculation and aphid-mediated inoculation with the potyvirus bean common mosaic virus (BCMV). Topical application of dsRNAs targeting either the coding region of the potyviral nuclear inclusion b (NIb) protein (BCMVNIb-dsRNA) or the coat protein (CP) coding region (BCMVCP-dsRNA) protected *Nicotiana benthamiana* and cowpea (*Vigna unguiculata*) plants against mechanical inoculation with BCMV. BCMVCP-dsRNA was selected for subsequent aphid transmission experiments. BCMVCP-dsRNA was loaded onto layered double hydroxide nanoparticles to form BCMVCP-BioClay which is a more stable formulation for delivering dsRNA than naked dsRNA. BCMVCP-BioClay was shown to be successful in protecting plants against BCMV transmission by the aphid *Myzus persicae*. Spraying detached *N. benthamiana* leaves with BCMVCP-BioClay 5 days prior to exposure to viruliferous aphids protected the leaves from infection by BCMV. Importantly, spraying of intact *N. benthamiana* and cowpea plants with BCMVCP-BioClay 5 days prior to exposure to viruliferous aphids protected plants of both species from BCMV infection. This study demonstrates that topical application of dsRNA using BioClay protects plants from aphid-mediated virus transmission, which is an important first step toward developing practical application of this approach in crop protection.

## Introduction

Bean common mosaic virus (BCMV) is a potyvirus that infects a wide range of wild and crop legumes and occurs worldwide (Worrall et al., 2015). Its most notable crop hosts are common bean (*Phaseolus vulgaris*), broad bean (*Vicia faba*), peanut/groundnut (*Arachis hypogaea*) and cowpea (*Vigna unguiculata*) (Morales and Bos, 1988; Bos, 1995). Symptoms of BCMV in these hosts include mosaic, leaf curling, dwarfing and chlorosis and in some cases necrosis (Flores-Estévez et al., 2003; Worrall et al., 2015). BCMV can be transmitted by mechanical inoculation (a common experimental approach), but in the field, it is transmitted through seed or, most importantly, by aphids, which spread the virus in a non-persistent manner (Silbernagel et al., 2001; Sastry, 2013). Aphids (Hemiptera: Aphididae) are phloem-feeding insects that have specialized needle-like mouthparts called stylets, which penetrate plant cells (Powell, 1993). Among the aphid species that transmit BCMV, the generalist aphid *Myzus persicae* is one of the most efficient vectors (Halbert et al., 1994; Worrall et al., 2015). Acquisition does not require prolonged feeding and non-persistently transmitted viruses are only loosely attached to the aphid stylet (Whitfield et al., 2015). Thus, for potyviruses, the processes of virus acquisition and virus inoculation both occur within 3–15 seconds of stylet penetration into plant epidermal cells (Powell, 1993).

Aphid-mediated transmission can rapidly spread BCMV. In Colombia, when aphid populations were reported at high levels, an initial BCMV prevalence of 2–6% due to seed contamination was magnified to 100% infection due to aphid-mediated transmission (Galvez and Morales, 1989). As a control measure, common bean cultivars utilizing the dominant *I* gene and recessive resistance genes have been specifically bred for BCMV resistance (Worrall et al., 2015). However, broad resistance to the different strains of BCMV and the closely related virus bean common mosaic necrosis virus (BCMNV) is difficult to achieve (Worrall et al., 2015). Control of the aphid vectors traditionally relies on insecticides and integrated pest management techniques (Westwood and Stevens, 2010). However, rising pesticide-resistant insect populations, concerns about associated health risks for farmers and consumers and the effects on beneficial insects (Florax et al., 2005) demonstrate the need for alternative protection methods. In any case, insecticides have little effectiveness in inhibiting transmission of non-persistently transmitted viruses like BCMV (Westwood and Stevens, 2010).

RNA interference (RNAi), also called RNA silencing, plays an important role in defence of many eukaryotic organisms against pathogens, especially against viruses (Carr et al., 2010; Csorba et al., 2015). In plants, dsRNA is processed stepwise into single-stranded small-interfering RNA molecules that are incorporated into an RNA-induced silencing complex, which degrades RNA homologous to the introduced dsRNA in a sequence-specific manner (Csorba et al., 2015). RNAi can be induced against viruses by expression in transgenic plants of constructs expressing dsRNA of various lengths ranging from shorter sizes of 50–150 base pairs (bp) and larger sizes of up to 2.5 kb (Pooggin, 2017). Many RNAi-inducing constructs have been designed against potyviruses such as: 1,048 bp targeted to the CP coding region of plum pox virus (Montes et al., 2014), a 302 bp construct targeting the P3 coding region of soybean mosaic virus (Yang et al., 2018), a 423 bp targeting the CP coding region of sorghum mosaic virus (Guo et al., 2015), an 899 bp hybrid construct targeted to 462 nucleotides (nt) of the CP coding region of cowpea aphid-borne mosaic virus, and a 415 nt construct targeting the proteinase cofactor coding region of cowpea severe mosaic virus (a comovirus) (Cruz and Aragão, 2013).

However, genetic transformation of some legume crop plants can be difficult or intractable. For example, only one transgenic *P. vulgaris* bean line has been reported, Embrapa 5.1, which protects against the geminivirus bean golden mosaic virus (Bonfim et al., 2007; ISAAA, 2018). An alternative approach to generate antiviral RNAi, especially in plants that are recalcitrant to transformation or regeneration, is the application of RNAi-inducing dsRNA molecules (Mitter et al., 2017b). Recently, it was shown that loading RNAi-inducing dsRNA into layered double hydroxide (LDH) nanoparticles (BioClay) and applying to plant surfaces by spraying facilitated sustained release of the dsRNA (Mitter et al., 2017a). This provided viral protection for up to 20 days post-treatment compared to the 5-day protection window generated by applying naked dsRNA (Mitter et al., 2017a) and marked a significant advance over previous methods used to induce antiviral RNAi in non-transgenic plants (Mitter et al., 2017b). In the previous study (Mitter et al., 2017a), resistance was assessed in plants treated with BioClay by viral challenge using mechanical inoculation. However, under field conditions, most plant viruses are transmitted by insect vectors (Groen et al., 2017; Carr et al., 2018). Therefore, in this study, we tested the efficacy of spraying BioClay carrying BCMV-specific dsRNA molecules in protection of cowpea and *N. benthamiana* plants against aphid-mediated transmission of BCMV.

## Materials and Methods

### Plant, Aphid and Virus Maintenance

Plants of *Nicotiana benthamiana* Domin. and *Vigna unguiculata* (L.) Walp. ssp. *unguiculata* cv. blackeye (cowpea) were grown in UC mix soil (Baker and Chandler, 1957) in 10 cm wide pots under glasshouse conditions at a mean temperature of 25°C under natural light. Plants were watered with a sprinkler system. For experiments with aphids, plants and detached leaves were placed in a 40 × 40 × 40 cm mesh aphid cage in a growth cabinet (Percival) at 26°C, 65% humidity and 16 h/8 h light/dark cycle. Under the same growth cabinet conditions, a colony of *Myzus persicae* (Sulzer) was maintained on *Capsicum annuum* L. plants. Aphids were starved before use to increase their virus transmission efficiency (Powell, 1993).

The Australian isolate BCMV 1 (GenBank accession MH220847) was revived (from freeze-dried leaf samples) and propagated in *N. benthamiana* (Worrall et al., 2019). Routine passaging and challenge of dsRNA-treated *N. benthamiana* and cowpea plants were conducted by mechanical inoculation using 50 μl sap from systemically infected *N. benthamiana* plants diluted 1:1500 in 0.01 M phosphate (pH 7.0) inoculation buffer with Carborundum used as an abrasive (Hamid et al., 2013).

Infection of *N. benthamiana* and cowpea with BCMV 1 was confirmed either by using the PathoScreen BCMV-specific antigen-coated plate (ACP) enzyme-linked immunosorbent assay (ELISA) kit (Agdia Inc., Elkhart Indiana) or by reverse transcription-coupled polymerase chain reaction (RT-PCR) assays. For RT-PCR, total RNA was extracted using TRIzol reagent (Thermo Fisher), and 1 μg RNA was reverse transcribed to cDNA using the Bioline SensiFAST cDNA kit and the cDNA amplified using MyTaq DNA Polymerase (Bioline) with the forward primer (5′-ATTCGCTGCATCATTTAGAGAG-3′) and reverse primer (5′-TTGCATTTTCAACCATTGGTT-3′). PCR conditions were: 95°C, 1 min; denaturation at 95°C, 15 s; annealing at 46°C for 15 s and extensions at 72°C for 10 s for 30 cycles. PCR products were visualized on a 1% agarose gel. The presence of a 962 bp band indicated detection of BCMV.

### Preparation of LDH and dsRNA

LDH nanosheets were prepared as previously described (Mitter et al., 2017a). Chemically synthesized dsRNA (Genolution Inc., Seoul, Republic of Korea) targeted to either 480 bp of the BCMV NIb coding region (7,865–8,344 nt GenBank accession MH220847) or 461 bp of the BCMV CP coding region (9,312–9,772 nt GenBank accession MH220847). BCMVNIb-dsRNA bioinformatic analysis of conserved motifs, mean pairwise percent identity and potential for 21 and 22 nt small-interfering RNAs was conducted as per Worrall et al. (2019). Double-stranded-RNA was received in a buffer solution and purified by adding an equal volume of 100% isopropanol and centrifuging at 16,200 *g* for 1 min. The pellet was washed twice with 70% ethanol before resuspending in sterile, RNase-free water. Optimal loading of dsRNA into LDH nanosheets was tested as described by Mitter et al. (2017a). Briefly, 250 ng BCMVNIb-dsRNA or BCMVCP-dsRNA was loaded onto different ratios of LDH nanosheets (i.e., dsRNA:LDH (w/w) was assayed at 1:0, 0:1, 1:1, 1:2, 1:3, 1:4, 1:5 and 1:10). Samples were incubated in a total volume of 12 μl at room temperature for 30 min. Loading profiles were assessed by gel electrophoresis with resolution on a 1% (w/v) agarose gel. The degree of dsRNA loading into the LDH nanosheets was assessed by the retention of dsRNA:LDH complexes in the well. All BioClay spray treatments were conducted using 1:2 dsRNA:LDH loading ratio, instead of the complete binding ratio of 1:4, to provide free dsRNA immediately upon spraying. Plants were sprayed with an atomizer (Mitter et al., 2017a).

### Determination of the Efficiency of Topically Applied Naked BCMVNIb-dsRNA and BCMVCP-dsRNA for RNAi-Mediated Viral Protection

Mechanical inoculation experiments were conducted with naked dsRNA to test the efficiency of the two types of BCMV-specific dsRNA. *N. benthamiana* plants at the six leaf stage were sprayed with 100 μg of GFP-dsRNA, BCMVNIb-dsRNA or BCMVCP-dsRNA in a 1 mL total volume of DEPC water per plant. The BCMV group was only virally challenged and did not receive a spray treatment. All plants were mechanically inoculated on a single leaf with 50 μl of a 1:2000 BCMV sap dilution in the inoculation buffer. Two 8-mm discs were collected from the two leaves closest to the plant apex at 10 days post-inoculation. Collected tissue was analysed by BCMV-specific ACP-ELISA as per manufacturer’s protocol (Agdia). Samples were considered positive when the mean value for absorbance (A^405^) exceeded by 3-fold the mean absorbance value from uninfected plants. Naked dsRNA protection studies were done with cowpea at the two-leaf stage. Cowpea was challenged on both leaves with 50 μl each of 1:1500 BCMV sap dilution. A total of 35 plants per group were tested for *N. benthamiana* over four experiments, and 30 per group were tested for cowpea over three experiments.

### Aphid-Mediated BCMV Transmission in Detached Leaf Assay

**Figure 1:**
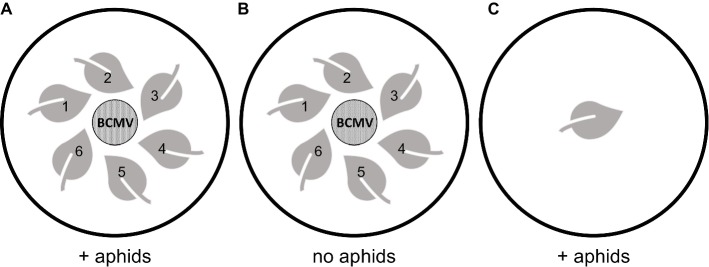
Experimental design of detached leaf aphid-mediated transmission assays. **(A)** set up for the treatment groups subjected to aphid infestation, six water or BCMVCP-BioClay treated leaves were placed around the BCMV leaf disc without contact, **(B)** set up of the control group (water treated leaves) without the addition of aphids to test for viral infection aside from aphid-mediated transmission and, **(C)** set up of the control group (water treated leaf) without a BCMV leaf disc source, but with the addition of aphids to test for virus-free aphids.

Aphid-mediated transmission of BCMV was tested in a detached leaf assay system. *N. benthamiana* seedlings (approx. 3 weeks old) in 15 cm pots were either untreated or sprayed with BCMVCP-BioClay (2 mL at 1:2 dsRNA:LDH loading ratio). Full leaves of the seedlings (approx. 1–1.5 cm diameter) were collected 5 days post-spraying. Six leaves were placed in a circle on 1% agar in 8 cm diameter Petri dishes (Figure 1A). Leaves were placed face up in a tight circle with the stem facing outwards. Care was taken to not let the leaves touch. Discs (15 mm diameter) were excised leaves of BCMV-infected *N. benthamiana* plants (infection having been authenticated by ELISA). The BCMV-infected leaf discs were placed in the centre of the circle, without touching the other leaves. Ten Petri dishes were used per treatment group. Aphids were starved overnight at 4°C. Thirty starved aphids were then placed batch wise in groups of 10 onto each BCMV-infected disc and confined on the infected disc for a minute before being allowed to roam (Figure 1A). Two control groups were included in each experiment to test for non-aphid-mediated transmission and any viral contamination of the aphids. To test for viral transmission from another source other than the aphids, three plates with a BCMV-infected disc and six untreated leaves did not receive aphids (Figure 1B). To test for viral contamination from the aphids, three dishes were set with an untreated leaf, and 30 aphids were added (Figure 1C). All Petri dishes were sealed with micropore tape and left at room temperature under natural light for 10 days. Each day, any aphids trapped in the media were collected and placed back onto the infected disc. At 10 days post-aphid infestation, all seven leaf discs were collected.

### Aphid-Mediated BCMV Transmission Seedling Experiments

**Figure 2:**
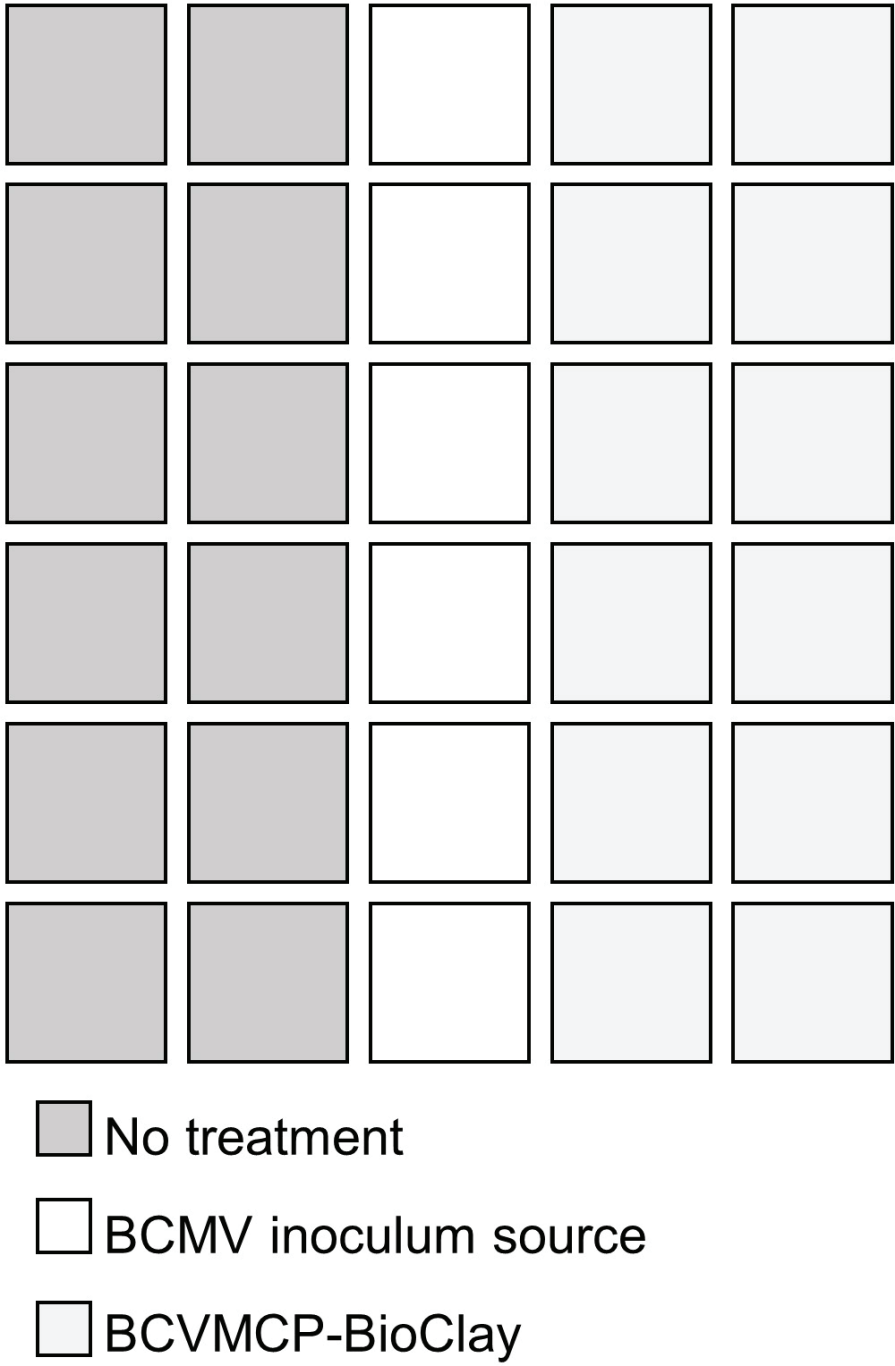
Experimental set up for aphid-transmission of BCMV on full seedlings of *N. benthamiana*. *N. benthamiana* seedlings newly at 4–6 leaf stage were either sprayed with 1:2 BCMVCP-BioClay or left untreated 5 days prior to aphid inoculation. Six plants in the middle were infected with BCMV as source plants. Thirty starved aphids were applied per BCMV source plant 5 days post-spraying. Plant tray was maintained in an insect cage in growth cabinet conditions for 20 days.

*N. benthamiana* seedlings were grown in a 30 (5 × 6) well tray and maintained in an insect cage under growth cabinet conditions. *N. benthamiana* plants at the 3–4 true leaf seedling stage were infected by mechanical inoculation with BCMV to serve as the inoculum source for virus acquisition by aphids (*n* = 6). The other *N. benthamiana* seedlings were sprayed with BCMVCP-BioClay (*n* = 12) or no-treatment (*n* = 12). The setup of the experiment is shown in Figure 2.

Thirty aphids were added to each of the BCMV source plants 5 days post-spraying and left to roam among the 30 plants for 20 days. Four 8-mm diameter leaf discs from individual leaves were collected at 10 and 20 days post-aphid placement and subjected to semi-quantitative PCR (details as described for leaf disc assays). The experiment was conducted in duplicate with *N. benthamiana.* The experiment was repeated with cowpea; however, five plants per group and two BCMV source plants were used due to size restraints of the tray. Cowpea plants were sprayed as soon as primary leaves formed. Samples were collected at 10 and 20 days post-aphid infestation and analysed as prior.

### Statistics

All statistical analysis was performed using custom R scripts executed in R studio (Crawley, 2012) using non-parametric Fisher’s exact test of independence with *post-hoc* Holm-Bonferroni multiple corrections.

## Results

### Double-Stranded RNA Synthesis and Choice of Target Sequences in the BCMV Genome

Recently, we designed and tested RNAi-inducing dsRNA molecules using transient expression *via* agroinfiltration in leaves of *Nicotiana benthamiana* (Worrall et al., 2019). Using two Australian BCMV isolates (BCMV 1 and 2), it was found that the most effective sequences to target in the BCMV genomic RNA for effective induction of RNAi-mediated resistance lie within the sequences encoding the nuclear inclusion b (NIb) protein and the CP (Worrall et al., 2019). To target these regions of the BCMV genome using BioClay, two BCMV-specific dsRNAs were synthesized. BCMVNIb-dsRNA was designed to target a 480 nt sequence within the BCMV NIb protein coding region and BCMVCP-dsRNA which targets 461 nt of the CP coding region. Due to the constraints of the dsRNA production, BCMVNIb-dsRNA (480 nt) was shorter than the 920 nt RNAi construct expressed in the transient assay experiments (Worrall et al., 2019). BCMVNIb-dsRNA was designed to target the sequence spanning 7,865–8,344 nt of the BCMV 1 genome. This 480 nt target sequence was inspected for mean pairwise percent identity and potential for generation of 21 and 22 nt short-interfering RNAs (Worrall et al., 2019). The mean pairwise percent identity of all 64 BCMV GenBank sequences and all 13 sequences of the closely related BCMNV aligned RNA sequences was 87.5% (see Supplementary Figure 1). These analyses suggest that BCMVNIb-dsRNA should induce antiviral RNAi as effectively as a longer 920 bp BCMVNIb-dsRNA tested by agroinfiltration (Worrall et al., 2019) (Supplementary Table 1). The BCMVCP-dsRNA target region was described in Worrall et al. (2019).

### Targeting the NIb and CP Sequences of BCMV With Exogenous dsRNA-Induced Resistance to the Virus

The effectiveness of the two dsRNAs, BCMVNIb-dsRNA and BCMVCP-dsRNA, for induction of resistance by topical application was tested in *N. benthamiana* and cowpea. Naked dsRNA was applied by spray onto plant foliage, and plants were subsequently challenged with BCMV using mechanical inoculation. Over four trials (*n* = 5, 10, 10 and 10 plants), *N. benthamiana* plants were sprayed at the six-leaf stage on day 0 and challenged on day 1. Groups included no treatment, BCMV only, GFP-dsRNA, BCMVNIb-dsRNA and BCMVCP-dsRNA. When analysed using BCMV-specific ELISA at 10 days post inoculation, 20% of BCMVNIb-dsRNA-treated and 6% of BCMVCP-dsRNA-treated plants were found to be systemically infected (Table 1). The positive control treatments, ‘BCMV only’ (no pre-treatment with dsRNA) and pre-treatment GFP-dsRNA, resulted in 77 and 57% of plants becoming infected with BCMV, respectively. The experiment was repeated on cowpea plants over three trials (*n* = 10, 10 and 10 plants); however, the cowpea plants were sprayed and challenged at the two-leaf stage. Similarly, BCMVNIb-dsRNA (47% infected) and BCMVCP-dsRNA (13% infected) showed RNAi-mediated protection when compared to ‘BCMV only’ (93% infected) and the non-specific, GFP-dsRNA (93% infected) (Table 1). The lack of protection observed in *N. benthamiana* and cowpea plants treated with the control dsRNA, GFP-dsRNA, demonstrated the specificity required for RNAi-mediated protection. The results obtained with *N. benthamiana* and cowpea demonstrate that topically applied naked BCMV-specific dsRNA can induce protection against BCMV infection in both hosts. Of the two dsRNAs, BCMVCP-dsRNA was the more effective and was therefore selected for subsequent experiments.

**Table 1:** Testing of dsRNA for protection against mechanical inoculation of BCMV.

Host	Treatment group	Infected^a^	Percent infected (%)	Total percent infected (%)
Cowpea	BCMV only	10/10	100	93
		9/10	90	
		9/10	90	
	GFP-dsRNA	15/15	100	93
		13/15	87	
	BCMVNIb-dsRNA	8/10	80	47^**^
		2/10	20	
		4/10	40	
	BCMVCP-dsRNA	1/10	10	13^***^
		1/10	10	
		2/10	20	
*N. benthamiana*	BCMV only	5/5	100	77
		8/10	80	
		5/10	50	
		9/10	90	
	GFP-dsRNA	5/5	100	57
		9/10	90	
		3/10	30	
		3/10	30	
	BCMVNIb-dsRNA	2/5	40	20^***^
		3/10	30	
		0/10	0	
		2/10	20	
	BCMVCP- dsRNA	1/5	20	6^***^
		0/10	0	
		2/10	20	
		0/10	0	

a *Infected as determined by ACP-ELISA* (Supplementary Figures 2–8).

^**^p < 0.01 and ^***^p < 0.001 statistical significance compared to BCMV only using Fisher’s exact test of independence with post-hoc Holm-Bonferroni multiple corrections.

Plants were sprayed with GFP-dsRNA (control treatment), BCMVNIb-dsRNA or BCMVCP-dsRNA and challenged with BCMV 1 day post-spraying. ‘BCMV only’ plants were untreated before challenging with BCMV. BCMV infection was determined using ELISA at 10 days post-inoculation. Total percent infected indicates the overall infection rate from two to four independent experiments per treatment.

### BioClay Treatment Protects Plants from Aphid-Mediated Inoculation with BCMV

**Figure 3:**
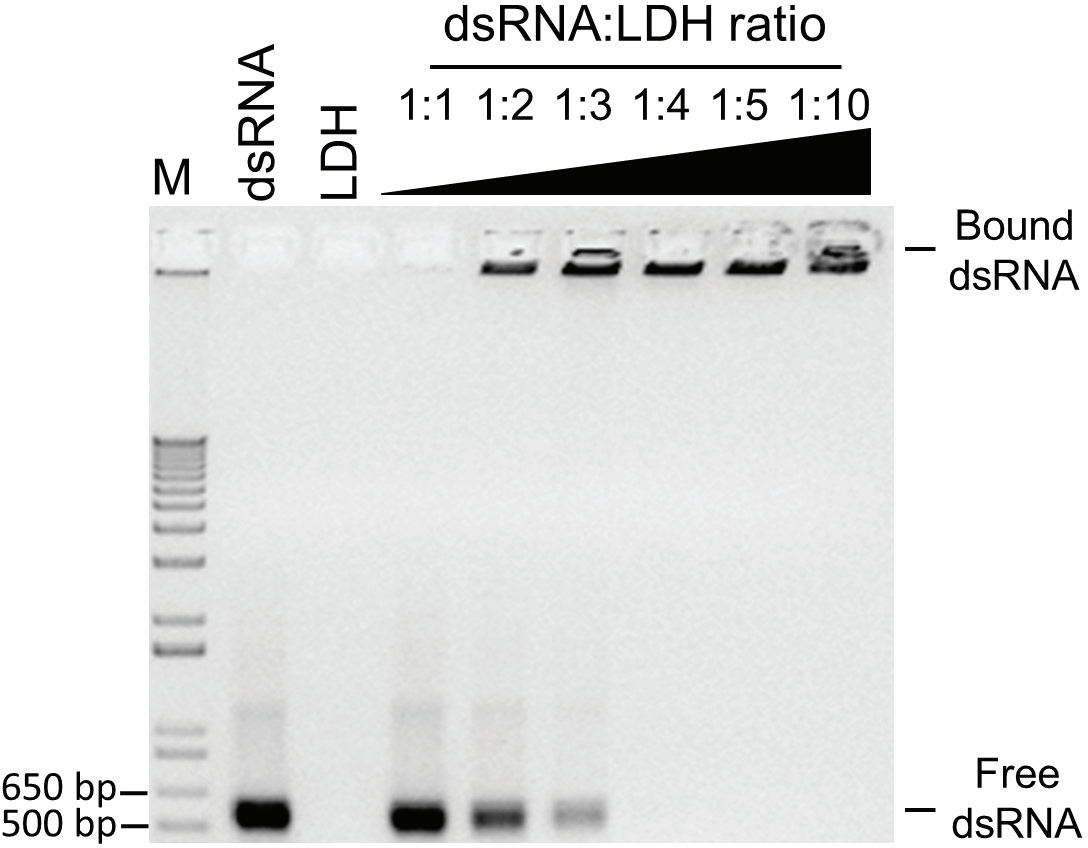
Loading profile of BCMVCP-dsRNA onto LDH nanosheets. DsRNA was loaded into LDH nanosheets using the mass ratios shown (dsRNA:LDH). BCMVCP-BioClay preparations (1:1-1:10), 1 kb + ladder (M), naked BCMVCP-dsRNA (dsRNA), and LDH (LDH) were subjected to agarose gel electrophoresis stained with ethidium bromide and imaged. LDH- bound dsRNA does not migrate and can be seen as fluorescence in the well while free dsRNA migrates through the agarose gel. Complete loading was achieved at a dsRNA-LDH mass ratio of 1:4 (lane 7).

Since exogenously applied BCMVCP-dsRNA provided the most effective protection, aphid-mediated transmission experiments were conducted with BCMVCP-dsRNA loaded onto LDH nanoparticles. Previous work showed that when the dsRNA was loaded onto the LDH nanoparticles, release of the dsRNA from the LDH occurred in a more sustained manner and provided a longer window of protection compared to naked dsRNA (Mitter et al., 2017a). BCMVCP-dsRNA was tested for loading capacity by loading onto the LDH nanoparticles in varying mass ratios with LDH (Figure 3). BCMVCP-dsRNA showed complete loading into LDH at a 1:4 dsRNA:LDH mass ratio (Figure 3). BCMV-specific dsRNA loaded onto LDH nanoparticles will be subsequently referred to as BCMVCP-BioClay.

To test if topical application of the BioClay preparation protected plant tissue from aphid-mediated inoculation with BCMV, a detached leaf assay was conducted. *N. benthamiana* seedlings were either sprayed with BCMVCP-BioClay or left untreated. At 5 days post-spraying, six leaves were detached from the stem of each seedling and placed on the surface of agar in Petri plates (Figure 1A). Leaf discs were excised from a BCMV-infected *N. benthamiana* plant (confirmed to be BCMV-infected by ELISA) and placed in the centre of the circle of leaves in each plate. Thirty starved aphids were placed directly onto each BCMV-infected leaf disc and released after a minute. Ten plates of leaves treated with BCMVCP-BioClay or untreated (*n* = 60 leaves) were used. Three plates were used as a no-aphid control to ensure no accidental mechanical inoculation of leaves occurred during handling. Three plates containing a single untreated leaf also received 30 starved aphids to check for any viral contamination coming from the aphid colony. Ten days after placement of aphids, the leaves were collected for extraction of total RNA and RT-PCR analysis using CP-specific primers.

The BCMVCP-BioClay-treated leaves (27/60) showed significant protection compared to untreated leaves (40/60) (^*^p < 0.05 significance: Fisher’s exact test of independence with *post-hoc* Holm-Bonferroni multiple corrections) (Supplementary Figure 9). No viral infection was observed in detached leaves in the absence of aphids (Figure 1B; Supplementary Figure 9). No virus was detected in the no-virus control plates (Figure 1C), demonstrating that the aphid colony was virus-free when introduced (Supplementary Figure 9). Thus, BCMVCP-BioClay protects detached *N. benthamiana* leaves when challenged with BCMV *via* aphid-mediated transmission.

**Figure 4:**
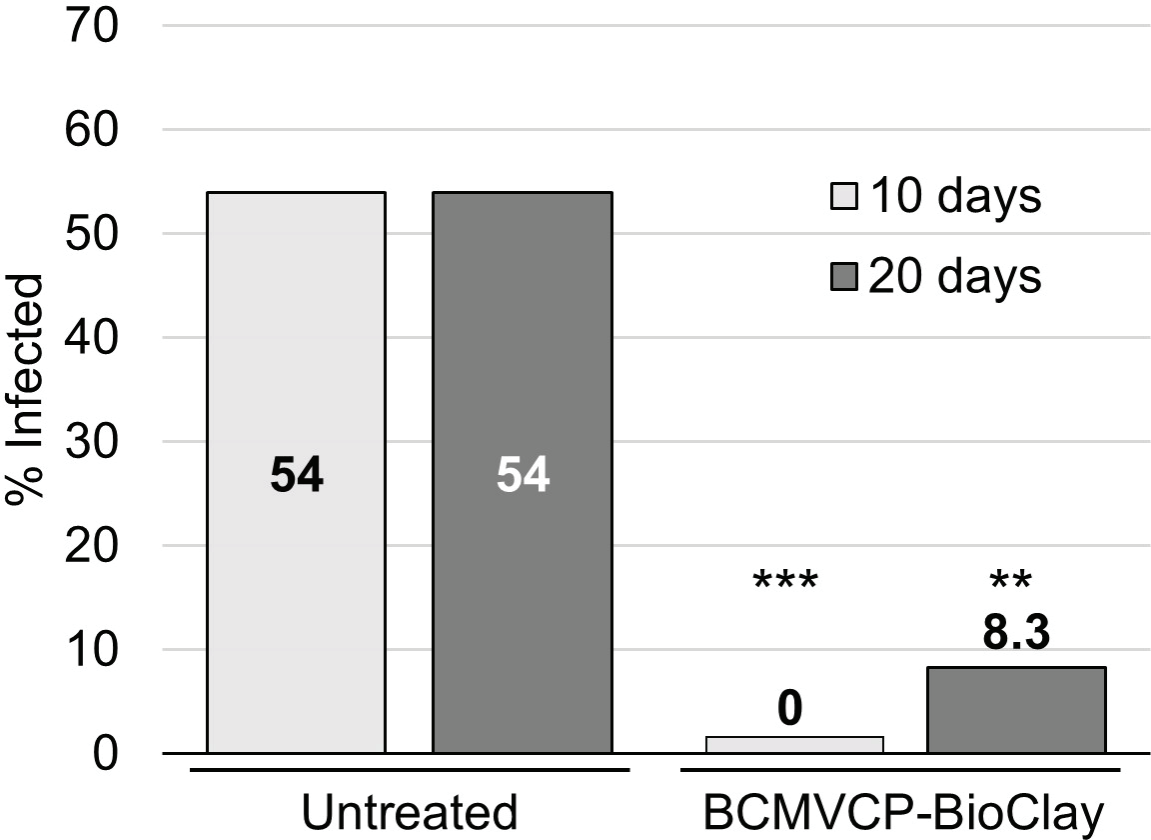
Spraying with BCMVCP-BioClay protected *Nicotiana benthamiana* plants from aphid-mediated transmission of BCMV. Over two experiments *N. benthamiana* seedlings were sprayed with BCMVCP-BioClay or left untreated (*n* = 12 plants per treatment group in each experiment). Five days later plants were exposed to aphids that were placed on BCMV-infected source plants. Leaf samples were collected at 10 and 20 days after exposure to aphids for detection of BCMV by RT-PCR. Statistical significance (^**^p < 0.01, ^***^p < 0.001) was determined using Fisher’s exact test of independence with *post-hoc* Holm-Bonferroni multiple corrections.

To determine if whole *N. benthamiana* plants could be protected from aphid-mediated inoculation and resulting systemic infection with BCMV, starved aphids were placed onto BCMV-infected plants arranged in a row between plants that were untreated or had been sprayed with BCMVCP-BioClay 5 days previously. Tissue for total RNA extraction was collected at 10 and 20 days after aphid infestation for RT-PCR to detect viral RNA. This experiment was repeated twice (Supplementary Figures 10 and 11). At 10 days post-aphid infestation over the two replicate trials, none of the 24 BCMVCP-BioClay-treated plants were infected, while 13 out of 24 untreated plants were positive for BCMV by PCR (^***^p < 0.001) (Figure 4). At 20 days post-infestation over the two replicate trials, only 2 out of 24 plants treated with BCMVCP-BioClay became BCMV-infected, while 13 out of 24 untreated plants became infected (^**^p < 0.01) (Figure 4).

**Figure 5:**
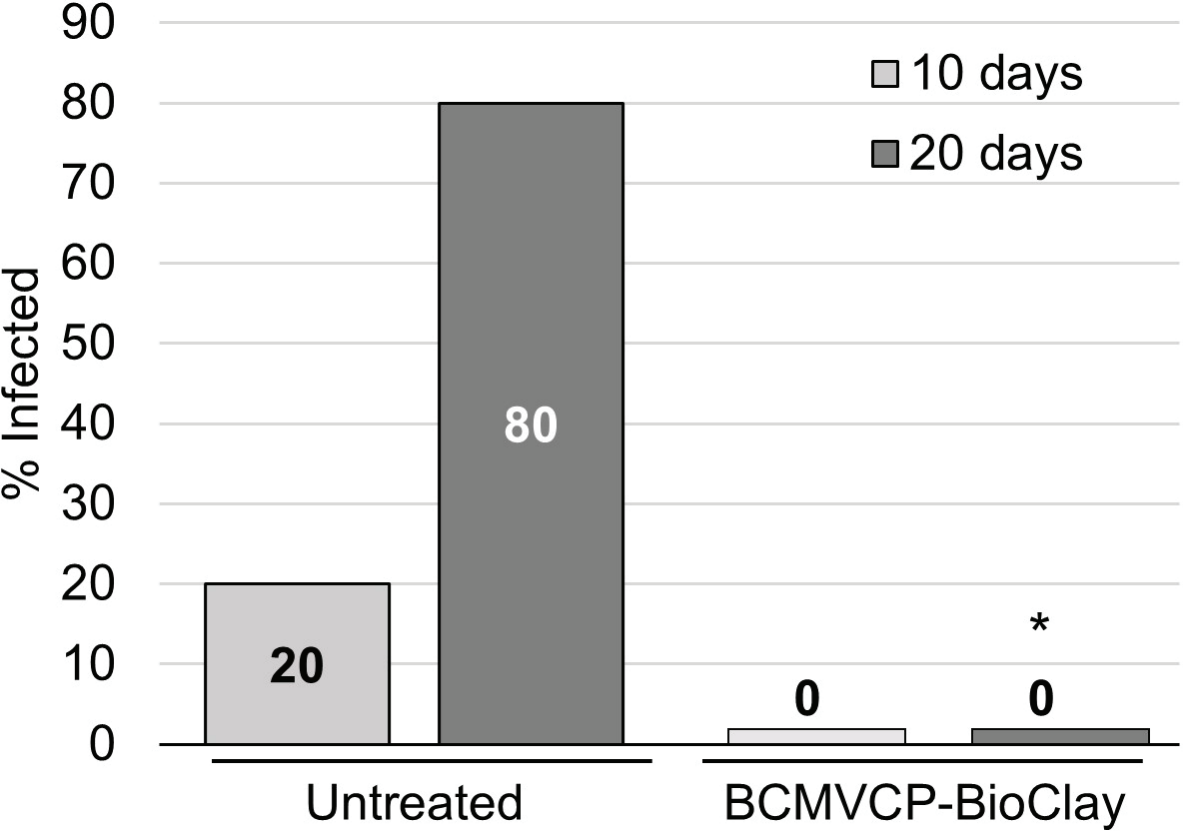
BCMVCP-BioClay protected cowpea plants from aphid-mediated transmission of BCMV. Cowpea plants were sprayed with BCMVCP-BioClay (*n* = 5) or left untreated (*n* = 5). Five days later plants were exposed to aphids from BCMV-infected plants. Leaf samples were collected at 10 and 20 days following exposure to aphids for extraction of RNA and detection of BCMV by RT-PCR. Statistical significance (^*^p < 0.05) was determined using Fisher’s exact test of independence with *post-hoc* Holm-Bonferroni multiple corrections.

Intact cowpea plants could also be protected against aphid-mediated BCMV infection following treatment with BCMVCP-BioClay. Samples were collected at 10 and 20 days following the introduction of the aphids. At 10 days post-aphid infestation, no plants treated with BCMVCP-BioClay became infected although only one out of five untreated plants showed symptoms of infection at that stage (no statistical significance) (Figure 5; Supplementary Figure 12). However, by 20 days following aphid infestation, four out of the five untreated plants were infected with BCMV (confirmed by RT-PCR), while no plants treated with BCMVCP-BioClay had become infected (^*^p < 0.05) (Figure 5; Supplementary Figure 12). Taken together, the experiments with detached leaves and intact plants of *N. benthamiana* and cowpea demonstrate that topical application of dsRNA-loaded BioClay can protect plants against aphid-borne virus infection.

## Discussion

For the topical application of dsRNA, two BCMV-specific dsRNAs were designed to target NIb and CP coding regions (BCMVNIb-dsRNA and BCMVCP-dsRNA, respectively). In a previous study, the antiviral RNAi-inducing efficiency of a BCMVNIb-dsRNA expressing construct and a BCMVCP-dsRNA expressing construct was confirmed by viral challenge using mechanical inoculation where 85 and 92% protection was observed, respectively (Worrall et al., 2019). However, in that study agroinfiltration—rather than topical application—was used to introduce T-DNAs expressing dsRNAs into the leaves of *N. benthamiana*. Another difference to the present study was that the BCMVNIb-dsRNA encoded by the T-DNA construct was 920 bp in length. However, due to constraints of dsRNA synthesis, it was not possible to generate a 920 bp molecule, and therefore, a 480 bp BCMVNIb-dsRNA was used in this study for exogenous application. Even at this reduced size, the 480 bp BCMVNIb-dsRNA provided protection against BCMV when exogenously applied to the foliage of *N. benthamiana* (80% protection) and cowpea (53% protection) (Table 1). The BCMVCP-dsRNA remained the same size in both studies and also provided protection against BCMV when it was topically applied (94 and 87% protection in *N. benthamiana* and cowpea, respectively) (Table 1). This shows that targeting these two regions of BCMV RNA to induce protection is an effective strategy using either agroinfiltration or topical application to introduce RNAi-inducing dsRNA molecules.

Tenllado and Díaz-Ruíz (2001) were the first to report antiviral protection by topical application of homologous dsRNA when it was mechanically co-inoculated with pepper mild mottle virus (PMMoV) onto a local lesion host. Plants that had been simultaneously inoculated with the virus and treated with a dsRNA did not exhibit PMMoV-induced lesions, unlike plants challenged with the virus alone (Tenllado and Díaz-Ruíz, 2001). Since then, protection using topical application of dsRNAs has been attempted against at least 11 other viruses and 3 viroids on at least 12 different plant hosts with varying degrees of efficacy (Mitter et al., 2017b). In Kaldis et al. (2017), dsRNA targeted to either 588 nt of the helper-component proteinase (HC-Pro) coding region or 498 nt of the CP coding region of zucchini yellow mosaic virus was exogenously applied to cucumber, watermelon and squash. Varied percentages of protection were observed on the respective host plants (HC-Pro: 18, 50 and 82% protection and CP: 30, 43 and 84% protection). This variability in protection on different hosts was also observed in the current study between the two BCMV hosts, cowpea (NIb 53% and CP 87% protection) and *N. benthamiana* (NIb 80% and CP 94% protection).

Although, topical application of naked dsRNA has been reported to be effective against viruses, fungi and insect pests (San Miguel and Scott, 2015; Koch et al., 2016; Wang et al., 2016; Ghosh et al., 2017), one of the key limitations is the instability of the dsRNA under environmental conditions, which would make it unsuitable for field use. A previous study showed that using LDH nanoparticles as carriers of dsRNA (BioClay) protects the dsRNA from degradation by nucleases, provides prolonged stability and also facilitates slow release of dsRNA on the plant surface (Mitter et al., 2017a). BioClay was shown to protect tobacco plants from the cucumovirus cucumber mosaic virus (CMV) and PMMoV (a tobamovirus) and cowpea plants from CMV when the viruses were introduced by mechanical inoculation (Mitter et al., 2017a). Furthermore, plants sprayed with BioClay were protected up to 20 days post-spraying compared to the 5-day protection afforded by naked dsRNA (Mitter et al., 2017a).

Insects transmit a majority of plant viruses, with aphids being the most common insect vectors (Groen et al., 2017; Carr et al., 2018). However, to the best of our knowledge, there is currently no report of successful protection of plants against vector-mediated virus transmission using topically applied dsRNA. This could be due to the relative ease of testing the efficiency of protection using mechanical inoculation compared to vector-mediated transmission. BCMV is a species within the Potyviridae, which is thought to be the largest grouping of plant viruses, including major pathogens of many important crops, of which most are vectored by insects (Revers and García, 2015; Worrall et al., 2015). BCMV is efficiently transmitted by several aphid species. For example, transmission rates in Sutter Pink bean leaves are 50% for *M. persicae*, 3% for *Metopolophium dirhodum*, 9.3% for *Rhopalosiphum padi*, 21.9% for *Schizaphis graminum* and 5% for *Sitobion avenae* (Halbert et al., 1994). The major advance in the current study is the optimisation and subsequent topical application studies with BCMV and *M. persicae* aphids as vectors. We used detached leaves and whole plants to test the efficiency of topically applied dsRNA, in the form of BioClay, for protection against aphid-transmitted BCMV. In the detached leaf assay, 45% of the BCMVCP-BioClay-treated leaves were infected, while 66.7% were infected with aphid-transmitted BCMV in the no treatment group. Progressing to whole plants, *N. benthamiana* plants sprayed with BCMVCP-BioClay were 8.3% infected, while 54% of untreated plants were positive for BCMV 20 days post-aphid infestation (Figure 4). Similar results were observed in cowpea plants where 0% of BCMVCP-BioClay plants were infected, while 80% of untreated plants succumbed to viral infection 20 days post-aphid infestation (Figure 5). The detached leaf assay with the *N. benthamiana* and cowpea plant studies show that BCMVCP-BioClay can protect plants from aphid-transmission of BCMV.

In this study, we have shown that exogenous application of dsRNA provides protection from viruses transmitted by aphids. This is significant because previous reports in the literature concerning topical application of RNAi-inducing dsRNAs used mechanical virus inoculation to challenge treated plants. This work represents an important proof of concept that BioClay can provide effective protection against the most common and important mode of virus transmission encountered under field conditions, a much needed step for practical application.

## Author Contributions

EW, JC, AB-C and NM wrote and edited the manuscript. EW, AB-C, KR, JC and NM planned aphid-transmission experiments. NM and JC provided expertise and supervised the work. EW performed mechanical inoculation experiments. EW and AN performed aphid-transmission experiments. SF conducted the bioinformatic analysis. EW performed statistical analysis.

### Conflict of Interest Statement

The authors declare that the research was conducted in the absence of any commercial or financial relationships that could be construed as a potential conflict of interest.
