# Supplementary material for: Exogenous Application of RNAi-Inducing Double-Stranded RNA Inhibits Aphid-Mediated Transmission of a Plant Virus
Source: Front Plant Sci. 2019 Mar 15;10:265. doi: 10.3389/fpls.2019.00265 (PMC6429036; doi:10.3389/fpls.2019.00265)
Supplement: Supplementary file 1 [file Table_1.docx]

Supplementary Material

Exogenous application of RNAi-inducing double-stranded RNA inhibits aphid-mediated transmission of a plant virus

Elizabeth A. Worrall^1^, Ana Bravo-Cazar^2^, Alexander T. Nilon^1^, Stephen J. Fletcher^1^, Karl E. Robinson^1^, John P. Carr^2^ and Neena Mitter^*1^

^1^Centre of Horticultural Science, Queensland Alliance of Agriculture and Food Innovation, The University of Queensland, Brisbane, QLD, Australia

^2^Department of Plant Sciences, Cambridge University, Cambridge CB2 3EA, U.K.

*** Correspondence:**

Neena Mitter
[n.mitter@uq.edu.au](mailto:n.mitter@uq.edu.au)


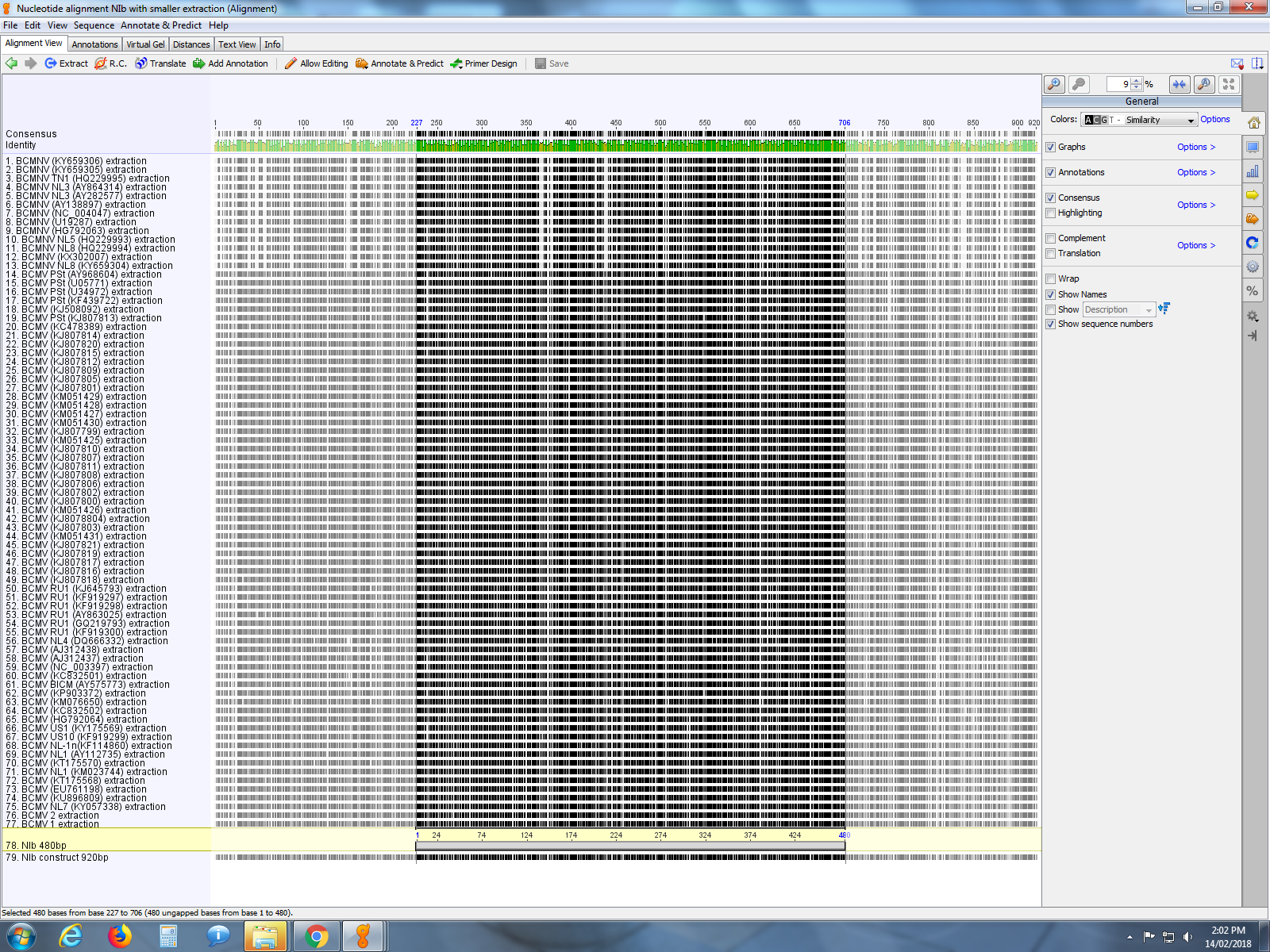


**Supplementary Figure 1 Alignment of BCMV and BCMNV sequences within the 480 bp NIb region targeted for RNAi-mediated resistance (showing previous 920 bp NIb region).** 62 BCMV and 13 BCMNV full genomic sequences from GenBank (accessed January 2018) were aligned with two Australian isolates (BCMV 1 and BCMV 2) and the previous 920 bp NIb construct and referenced against the new, highlighted 480 bp NIb dsRNA construct (bottom). The consensus identity (top) shows 100% preservation of nt sequence across all 75 sequences in green, varying degrees of nt conservation in yellow (higher is more conserved) and very low conservation in red. The consensus of the 480 bp region is highly conserved. Individual sequences show high conservation as indicated by the presence of black shading. The 13 BCMNV sequences (first 13 sequences) show less conservation towards the NIb target dsRNA, while the BCMV sequences show higher conservation. The pairwise identity of all aligned sequences was 85.1% for the 920 bp construct and 87.5% for the 480 bp construct.

­**Supplementary Table 1** Comparison of the number of 21 nt and 22 nt potential siRNA hits generated from the designed dsRNA targeted regions between the 920 bp and 480 bp BCMVNIb-dsRNA targeted regions, against the individual sequences corresponding to the 62 BCMV and 13 BCMNV GenBank sequences (accessed January 2018).

|  | **GenBank Sequence** | **NIb target (920 bp)** | | **NIb target (480 bp)** | |
| --- | --- | --- | --- | --- | --- |
|  |  | **21 nt hits** | **22 nt hits** | **21 nt hits** | **22 nt hits** |
| **BCMV Cluster 1a** | BCMV (KU896809) | 816 | 811 | 411 | 408 |
|  | BCMV (KT175568) | 791 | 785 | 397 | 393 |
|  | BCMV (EU761198) | 732 | 725 | 403 | 400 |
|  | BCMV NL7 (KY057338) | 816 | 811 | 432 | 430 |
|  | BCMV NL1 (KM023744) | 597 | 583 | 334 | 327 |
|  | BCMV NL-1n(KF114860) | 513 | 499 | 229 | 221 |
|  | BCMV NL1 (AY112735) | 606 | 593 | 316 | 309 |
|  | BCMV (KT175570) | 643 | 631 | 353 | 347 |
|  | BCMV (HG792064) | 478 | 472 | 273 | 268 |
|  | BCMV US10 (KF919299) | 620 | 610 | 323 | 318 |
|  | BCMV US1 (KY175569) | 565 | 552 | 331 | 326 |
|  | BCMV (KM076650) | 109 | 99 | 45 | 42 |
|  | BCMV (KP903372) | 103 | 96 | 66 | 62 |
| **BCMV Cluster 2** | BCMV (KC832502) | 145 | 133 | 62 | 55 |
|  | BCMV (AJ312438) | 173 | 163 | 86 | 82 |
|  | BCMV (AJ312437) | 142 | 135 | 104 | 99 |
|  | BCMV (NC_003397) | 142 | 135 | 104 | 99 |
|  | BCMV (KC832501) | 109 | 101 | 64 | 61 |
|  | BCMV BICM (AY575773) | 134 | 126 | 79 | 76 |
|  | BCMV NL4 (DQ666332) | 36 | 33 | 6 | 5 |
|  | BCMV RU1 (KJ645793) | 43 | 38 | 15 | 13 |
|  | BCMV RU1 (KF919297) | 18 | 15 | 15 | 13 |
|  | BCMV RU1 (KF919298) | 18 | 15 | 15 | 13 |
|  | BCMV RU1 (AY863025) | 22 | 19 | 15 | 13 |
|  | BCMV RU1 (GQ219793) | 35 | 32 | 15 | 13 |
|  | BCMV RU1 (KF919300) | 35 | 32 | 15 | 13 |
| **BCMV Cluster 1b** | BCMV (KM051428) | 46 | 40 | 23 | 18 |
|  | BCMV (KM051427) | 46 | 40 | 23 | 18 |
|  | BCMV (KM051429) | 46 | 40 | 23 | 18 |
|  | BCMV (KM051430) | 46 | 40 | 23 | 18 |
|  | BCMV (KM051425) | 46 | 40 | 23 | 18 |
|  | BCMV (KJ807799) | 46 | 40 | 23 | 18 |
|  | BCMV (KJ807801) | 63 | 56 | 34 | 29 |
|  | BCMV (KJ807805) | 42 | 38 | 19 | 16 |
|  | BCMV (KJ807812) | 45 | 40 | 22 | 18 |
|  | BCMV (KJ807810) | 57 | 51 | 34 | 29 |
|  | BCMV (KJ807809) | 51 | 45 | 28 | 23 |
|  | BCMV (KJ807811) | 42 | 38 | 19 | 16 |
|  | BCMV (KJ807807) | 45 | 40 | 22 | 18 |
|  | BCMV (KJ8078804) | 45 | 40 | 22 | 18 |
|  | BCMV (KJ807806) | 57 | 51 | 34 | 29 |
|  | BCMV (KM051431) | 60 | 55 | 22 | 18 |
|  | BCMV (KJ807803) | 57 | 53 | 19 | 16 |
|  | BCMV (KM051426) | 63 | 56 | 34 | 29 |
|  | BCMV (KJ807800) | 63 | 56 | 34 | 29 |
|  | BCMV (KJ807802) | 63 | 56 | 34 | 29 |
|  | BCMV (KJ807808) | 45 | 40 | 22 | 18 |
|  | BCMV (KJ807815) | 37 | 33 | 17 | 14 |
|  | BCMV (KJ807814) | 56 | 51 | 21 | 19 |
|  | BCMV (KJ807818) | 70 | 64 | 23 | 20 |
|  | BCMV (KJ807817) | 52 | 47 | 23 | 20 |
|  | BCMV (KJ807816) | 62 | 55 | 23 | 20 |
|  | BCMV (KJ807819) | 61 | 57 | 22 | 20 |
|  | BCMV (KJ807821) | 55 | 50 | 22 | 20 |
|  | BCMV (KC478389) | 37 | 31 | 25 | 20 |
|  | BCMV (KJ807820) | 58 | 52 | 22 | 20 |
|  | BCMV PSt (KJ807813) | 65 | 59 | 30 | 26 |
|  | BCMV (KJ508092) | 78 | 72 | 43 | 39 |
|  | BCMV PSt (KF439722) | 78 | 72 | 43 | 39 |
|  | BCMV PSt (U34972) | 81 | 74 | 46 | 41 |
|  | BCMV PSt (U05771) | 81 | 74 | 46 | 41 |
|  | BCMV PSt (AY968604) | 27 | 24 | 22 | 20 |
| **BCMNV cluster** | BCMNV (NC_004047) | 0 | 0 | 0 | 0 |
|  | BCMNV (AY138897) | 0 | 0 | 0 | 0 |
|  | BCMNV (U19287) | 0 | 0 | 0 | 0 |
|  | BCMNV NL3 (AY282577) | 0 | 0 | 0 | 0 |
|  | BCMNV NL5 (HQ229993) | 3 | 2 | 3 | 2 |
|  | BCMNV (HG792063) | 0 | 0 | 0 | 0 |
|  | BCMNV TN1 (HQ229995) | 0 | 0 | 0 | 0 |
|  | BCMNV (KY659305) | 0 | 0 | 0 | 0 |
|  | BCMNV (KY659306) | 0 | 0 | 0 | 0 |
|  | BCMNV NL3 (AY864314) | 0 | 0 | 0 | 0 |
|  | BCMNV NL8 (KY659304) | 0 | 0 | 0 | 0 |
|  | BCMNV (KX302007) | 0 | 0 | 0 | 0 |
|  | BCMNV NL8 (HQ229994) | 0 | 0 | 0 | 0 |

**
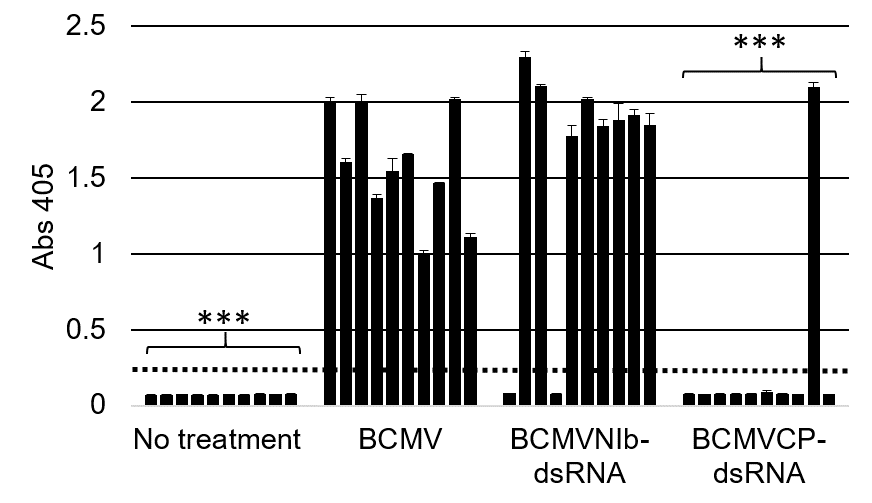
**

**Supplementary Figure 2** **BCMV infection on dsRNA sprayed cowpea plants when challenged with the virus 1 day post treatment (trial 1).** Plants were sprayed on day 0 and inoculated with BCMV on sprayed leaves 1 day post treatment. ACP-ELISA absorbance readings of the two most apical leaves 10 days pvc. Treatments included; no treatment (n=10), BCMV (n=10), BCMVNIb-dsRNA (n=10) and BCMVCP-dsRNA (n=10). Columns represent the average of two wells per sample ± standard error. BCMV positive threshold is ≥ three times the average ACP-ELISA absorbance reading of the no treatment controls, shown by the dotted line (positive threshold ≥0.221). 0/10 no treatment plants tested positive for BCMV, 10/10 BCMV treated plants tested positive for BCMV, 8/10 BCMVNIb-dsRNA treated plants tested positive for BCMV and 1/10 BCMVCP-dsRNA treated plants tested positive for BCMV. ^***^P<0.001 significance using Fisher’s exact test of independence with post-hoc Holm-Bonferroni multiple correction when compared to BCMV group.


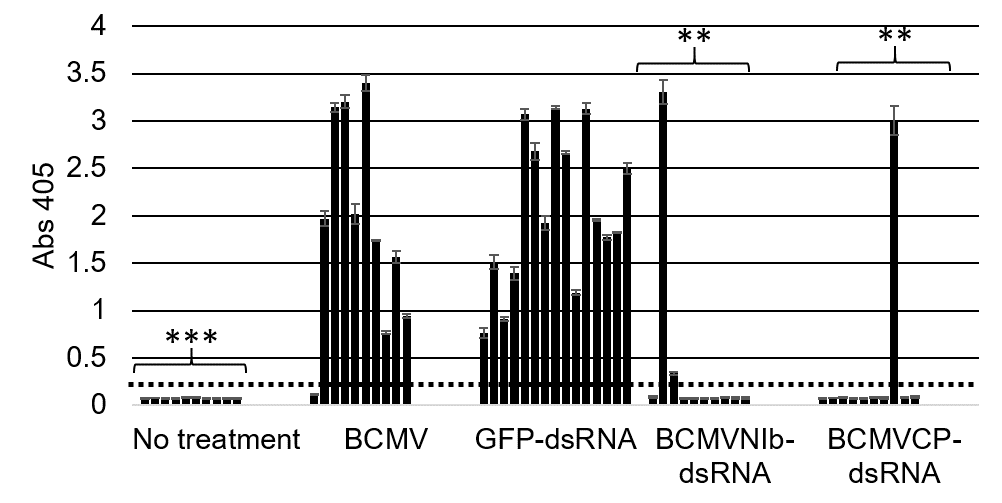


**Supplementary Figure 3** **BCMV infection on dsRNA sprayed cowpea plants when challenged with the virus 1 day post treatment (trial 2).** Plants were sprayed on day 0 and inoculated with BCMV on sprayed leaves 1 day post treatment. ACP-ELISA absorbance readings of the two most apical leaves 10 days pvc. Treatments included; no treatment (n=10), BCMV (n=10), GFP-dsRNA (n=15), BCMVNIb-dsRNA (n=10) and BCMVCP-dsRNA (n=10). Columns represent the average of two wells per sample ± standard error. BCMV positive threshold is ≥ three times the average ACP-ELISA absorbance reading of the no treatment controls, shown by the dotted line (positive threshold ≥0.230). 0/10 no treatment plants tested positive for BCMV, 9/10 BCMV treated plants tested positive for BCMV, 15/15 GFP-dsRNA treated plants tested positive for BCMV, 2/10 BCMVNIb-dsRNA treated plants tested positive for BCMV and 1/10 BCMVCP-dsRNA treated plants tested positive for BCMV. ^**^P<0.01, ^***^P<0.001 significance using Fisher’s exact test of independence with post-hoc Holm-Bonferroni multiple correction when compared to BCMV group.


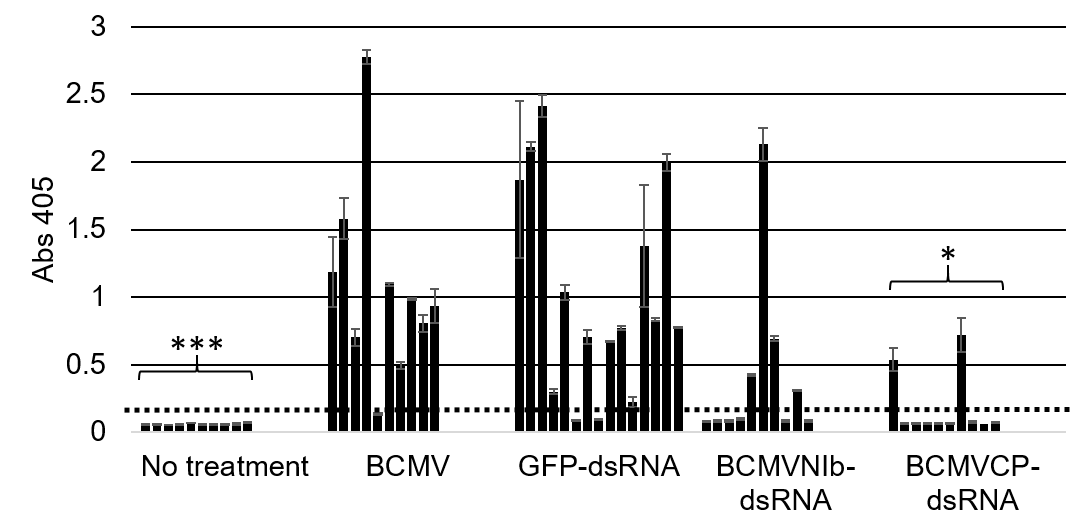


**Supplementary Figure 4** **BCMV infection on dsRNA sprayed cowpea plants when challenged with the virus 1 day post treatment (trial 3).** Plants were sprayed on day 0 and inoculated with BCMV on sprayed leaves 1 day post treatment. ACP-ELISA absorbance readings of the two most apical leaves 10 days pvc. Treatments included; no treatment (n=10), BCMV (n=10), GFP-dsRNA (n=15), BCMVNIb-dsRNA (n=10) and BCMVCP-dsRNA (n=10). Columns represent the average of two wells per sample ± standard error. BCMV positive threshold is ≥ three times the average ACP-ELISA absorbance reading of the no treatment controls, shown by the dotted line (positive threshold ≥0.188). 0/10 no treatment plants tested positive for BCMV, 9/10 BCMV treated plants tested positive for BCMV, 13/15 GFP-dsRNA treated plants tested positive for BCMV, 4/10 BCMVNIb-dsRNA treated plants tested positive for BCMV and 2/10 BCMVCP-dsRNA treated plants tested positive for BCMV. ^*^P<0.05, ^***^P<0.001 significance using Fisher’s exact test of independence with post-hoc Holm-Bonferroni multiple correction when compared to BCMV group.


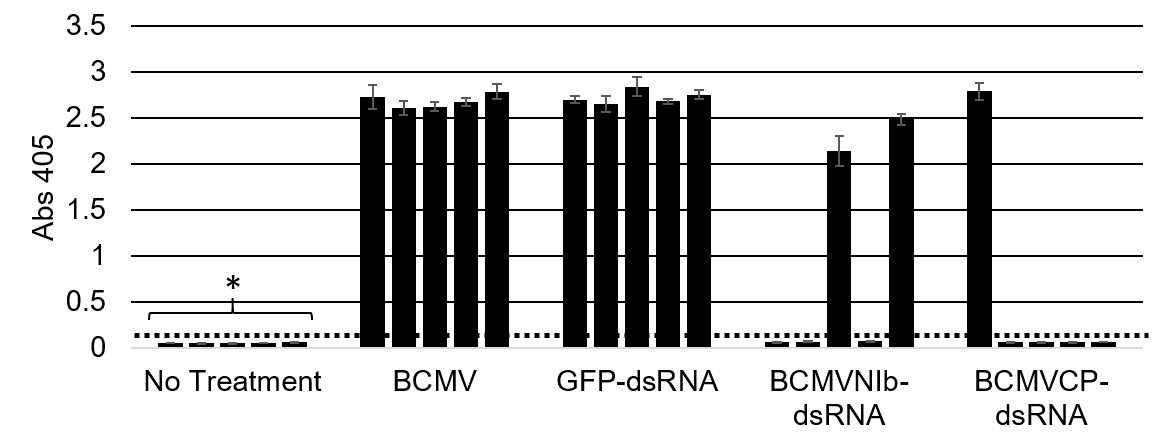


**Supplementary Figure 5** **BCMV infection on dsRNA sprayed *N. benthamiana* plants when challenged with the virus 1 day post treatment (trial 1).** Plants were sprayed on day 0 and inoculated with BCMV on sprayed leaves 1 day post treatment. ACP-ELISA absorbance readings of the two most apical leaves 10 days pvc. Treatments included; no treatment (n=5), BCMV (n=5), GFP-dsRNA (n=5), BCMVNIb-dsRNA (n=5) and BCMVCP-dsRNA (n=5). Columns represent the average of two wells per sample ± standard error. BCMV positive threshold is ≥ three times the average ACP-ELISA absorbance reading of the no treatment controls, shown by the dotted line (positive threshold ≥0.166). 0/5 no treatment plants tested positive for BCMV, 5/5 BCMV treated plants tested positive for BCMV, 5/5 GFP-dsRNA treated plants tested positive for BCMV, 2/5 BCMVNIb-dsRNA treated plants tested positive for BCMV and 2/5 BCMVCP-dsRNA treated plants tested positive for BCMV. ^*^P<0.05, significance using Fisher’s exact test of independence with post-hoc Holm-Bonferroni multiple correction when compared to BCMV group.


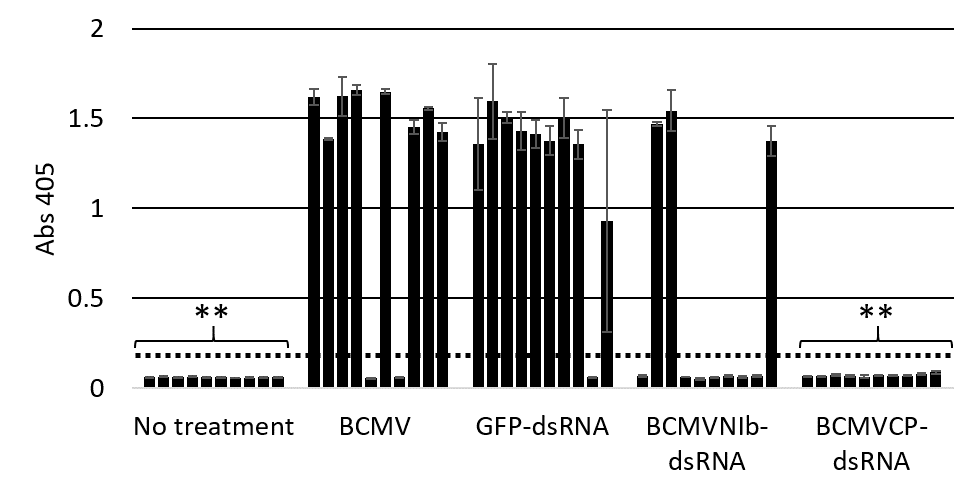


**Supplementary Figure 6** **BCMV infection on dsRNA sprayed *N. benthamiana* plants when challenged with the virus 1 day post treatment (trial 2).** Plants were sprayed on day 0 and inoculated with BCMV on sprayed leaves 1 day post treatment. ACP-ELISA absorbance readings of the two most apical leaves 10 days pvc. Treatments included; no treatment (n=10), BCMV (n=10), GFP-dsRNA (n=10), BCMVNIb-dsRNA (n=10) and BCMVCP-dsRNA (n=10). Columns represent the average of two wells per sample ± standard error. BCMV positive threshold is ≥ three times the average ACP-ELISA absorbance reading of the no treatment controls, shown by the dotted line (positive threshold ≥0.184). 0/10 no treatment plants tested positive for BCMV, 8/10 BCMV treated plants tested positive for BCMV, 9/10 GFP-dsRNA treated plants tested positive for BCMV, 3/10 BCMVNIb-dsRNA treated plants tested positive for BCMV and 0/10 BCMVCP-dsRNA treated plants tested positive for BCMV. ^**^P<0.01 significance using Fisher’s exact test of independence with post-hoc Holm-Bonferroni multiple correction when compared to BCMV group.

**
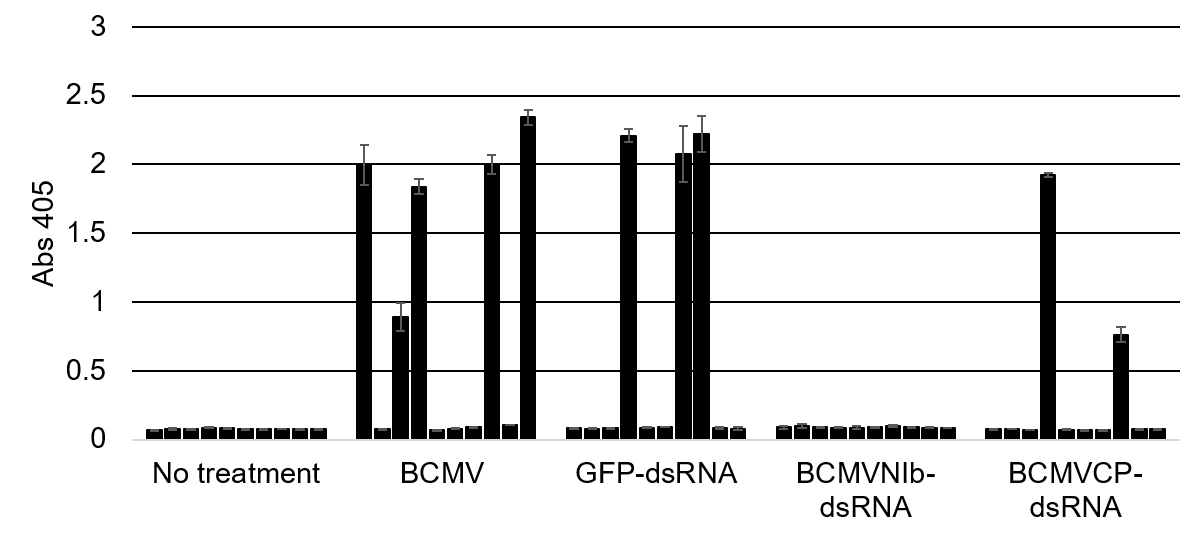
**

**Supplementary Figure 7** **BCMV infection on dsRNA sprayed *N. benthamiana* plants when challenged with the virus 1 day post treatment (trial 3).** Plants were sprayed on day 0 and inoculated with BCMV on sprayed leaves 1 day post treatment. ACP-ELISA absorbance readings of the two most apical leaves 10 days pvc. Treatments included; no treatment (n=10), BCMV (n=10), GFP-dsRNA (n=10), BCMVNIb-dsRNA (n=10) and BCMVCP-dsRNA (n=10). Coloumns represent the average of two wells per sample ± standard error. BCMV positive threshold is ≥ three times the average ACP-ELISA absorbance reading of the no treatment controls, shown by the dotted line (positive threshold ≥0.234). 0/10 no treatment plants tested positive for BCMV, 5/10 BCMV treated plants tested positive for BCMV, 3/10 GFP-dsRNA treated plants tested positive for BCMV, 0/10 BCMVNIb-dsRNA treated plants tested positive for BCMV and 2/10 BCMVCP-dsRNA treated plants tested positive for BCMV. No significance using Fisher’s exact test of independence with post-hoc Holm-Bonferroni multiple correction when compared to BCMV group.


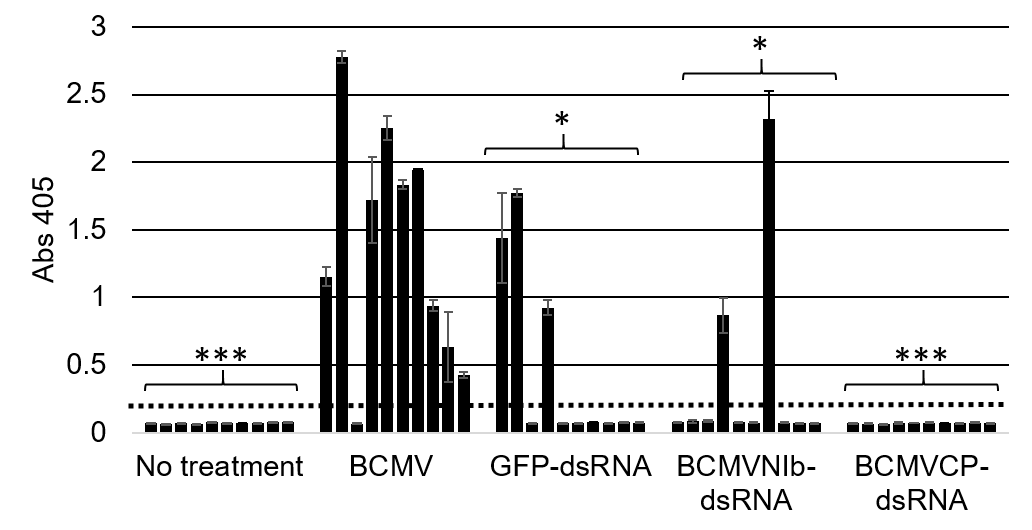


**Supplementary Figure 8** **BCMV infection on dsRNA sprayed *N. benthamiana* plants when challenged with the virus 1 day post treatment (trial 4).** Plants were sprayed on day 0 and inoculated with BCMV on sprayed leaves 1 day post treatment. ACP-ELISA absorbance readings of the two most apical leaves 10 days pvc. Treatments included; no treatment (n=10), BCMV (n=10), GFP-dsRNA (n=10), BCMVNIb-dsRNA (n=10) and BCMVCP-dsRNA (n=10). Columns represent the average of two wells per sample ± standard error. BCMV positive threshold is ≥ three times the average ACP-ELISA absorbance reading of the no treatment controls, shown by the dotted line (positive threshold ≥0.217). 0/10 no treatment plants tested positive for BCMV, 9/10 BCMV treated plants tested positive for BCMV, 3/10 GFP-dsRNA treated plants tested positive for BCMV, 2/10 BCMVNIb-dsRNA treated plants tested positive for BCMV and 0/10 BCMVCP-dsRNA treated plants tested positive for BCMV. ^*^P<0.05, ^***^P<0.001 significance using Fisher’s exact test of independence with post-hoc Holm-Bonferroni multiple correction when compared to BCMV group.

**
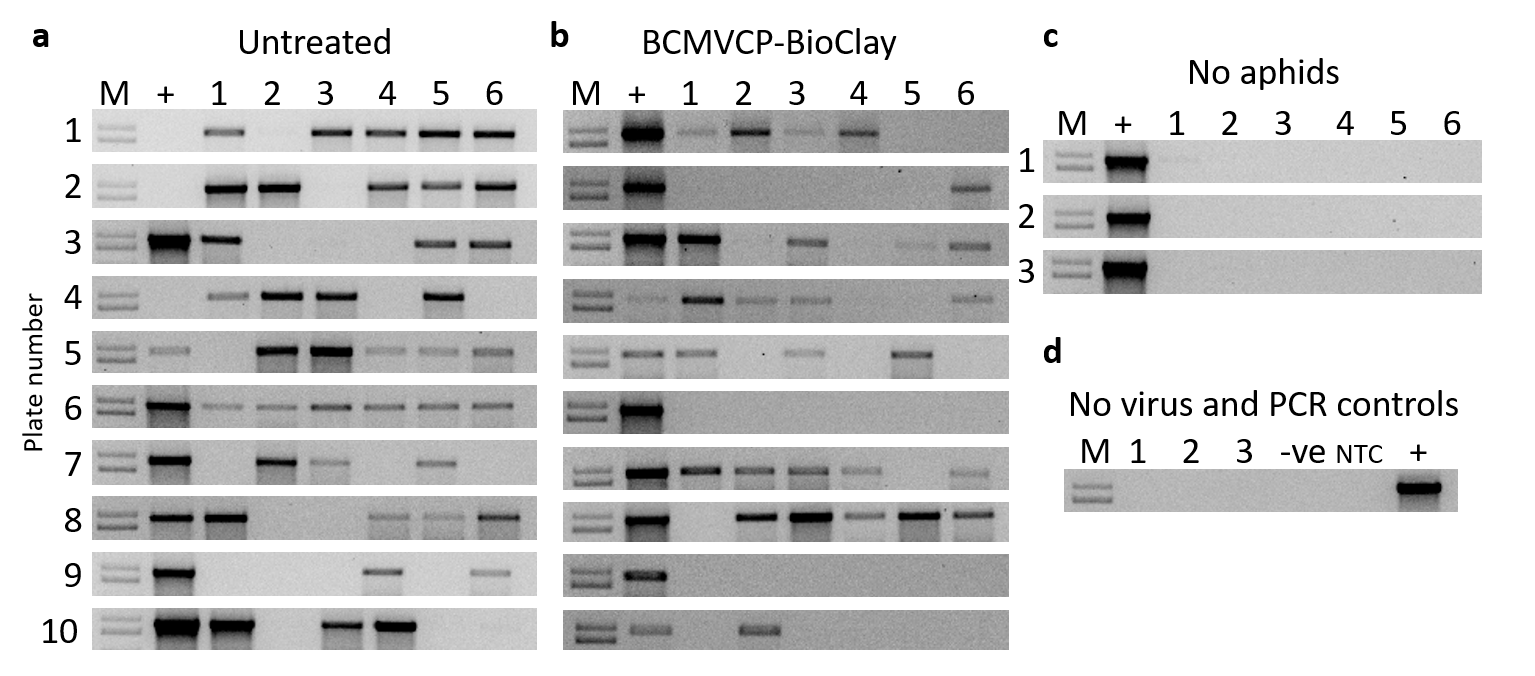
**

**Supplementary Figure 9 BCMV infection in *N. benthamiana* leaf discs when transmitted by aphids 5 days post spray treatment.** Detection of BCMV by Reverse-transcription PCR (962 bp). *N. benthamiana* leaves were **a)** untreated (n=60) or **b)** sprayed with BioClay 5 days prior to aphid infection (n=60). BCMV infected leaf disc (+) was placed in the center of 1% agar petri dish (10 dishes per group) and 6 leaves (lanes 1-6) were placed in a circle around the infected disc. 30 aphids were placed onto the infected disc. Samples collected 10 days post aphid-transmission (note: Untreated 1, 2, 4 and BioClay 4 BCMV discs were rotten). **a)** BCMV infection in untreated leaves was 40 out of 60 (66.7%) and **b)** 27 out of 60 leaves (45%) were infected in BioClay treated leaves. **c)** BCMV disc with untreated leaves without aphids (n=18). No mechanical inoculation occurred. **d)** Virus-free leaf disc with 30 aphids (n=3). No viral transmission occurred without BCMV infected tissue. PCR controls shown: uninfected *N. benthamiana* (-ve), no template (NTC) and BCMV-infected stock plant (+). M = 1kb+ ladder (850 bp and 1kb bp shown).


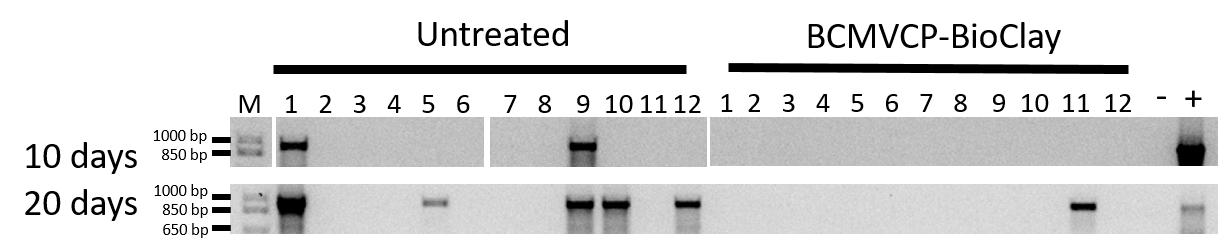


**Supplementary Figure 10 BioClay trial with aphid-mediated transmission of BCMV using *N. benthamiana* seedlings in soil (Trial 1).** *N. benthamiana* seedlings grown in soil were either sprayed with BCMVCP-BioClay or left untreated on day 0 and BCMV infected plants were infected with aphids 5 days post spray. Aphids were left to roam and samples were collected 10 and 20 days post aphid infection. At 10 days, 2/12 plants were infected with BCMV in the non-treated group and 0/12 plants in the BCMVCP-BioClay treated group. At 20 days, 5/12 plants were infected with BCMV in the non-treated group and 1/12 plants in the BCMVCP-BioClay treated group. Infection determined by the presence of a 962 bp band. – and + are controls of PCR.

**
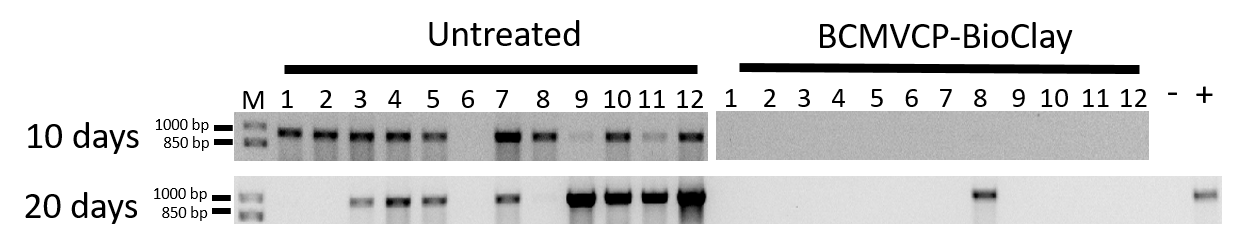
**

**Supplementary Figure 11 BioClay trial with aphid-mediated transmission of BCMV using *N. benthamiana* seedlings in soil (Trial 2).** *N. benthamiana* seedlings grown in soil were either sprayed with BCMVCP-BioClay or left untreated on day 0 and BCMV infected plants were infected with aphids 5 days post spray. Aphids were left to roam and samples were collected 10 and 20 days post aphid infection. At 10 days, 11/12 plants were infected with BCMV in the non-treated group and 0/12 plants in the BCMVCP-BioClay treated group. At 20 days, 8/12 plants were infected with BCMV in the non-treated group and 1/12 plants in the BCMVCP-BioClay treated group. Infection determined by the presence of a 962 bp band. – and + are controls of PCR.


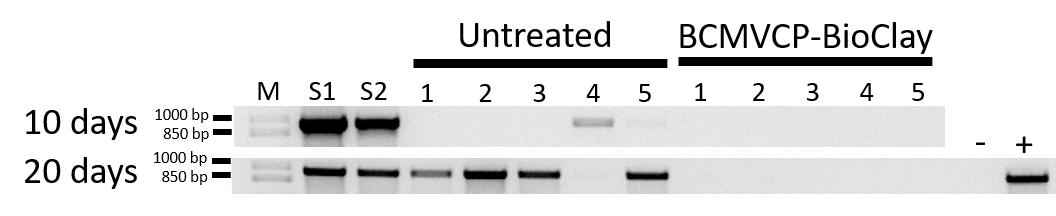


**Supplementary Figure 12** **BioClay trial with aphid-mediated transmission of BCMV using cowpea plants in soil.** Cowpea grown in soil were sprayed with either nothing (non-treated) or BCMVCP-BioClay on day 0 and BCMV infected plants were infected with aphids 5 days post spray. Aphids were left to roam and samples were collected 10 and 20 days post aphid infection. At 10 days, 1/5 plants were infected with BCMV in the non-treated group and 0/5 plants in the BCMVCP-BioClay treated group. At 20 days, 4/5 plants were infected with BCMV in the non-treated group and 0/5 in the BCMVCP-BioClay treated group. Infection determined by the presence of a 962 bp band. – and + are controls of PCR.
